# Effects on physical activity, physical fitness and well-being in a 36-months randomized controlled study, comparing a multimodal hospital-based intervention programme for primary cardiovascular prevention with usual care

**DOI:** 10.1186/s12872-024-03892-1

**Published:** 2024-04-25

**Authors:** Hilde Bergum, Jostein Grimsmo, Sigmund Alfred Anderssen, Tor Ole Klemsdal

**Affiliations:** 1 Department of Cardiac and Pulmonary Rehabilitation, Lovisenberg Rehabilitation, Cathinka Guldbergs Hospital, Jessheim, 2051 Norway; 2 University of Oslo, Oslo, Norway; 3 Department of Sports Medicine, the Norwegian School of Sports Sciences, Sognsveien 220, 0806 Oslo, Norway; 4 Department of Preventive Cardiology, Oslo University Hospital Aker, Trondheimsveien 235, 0586 Oslo, Norway

**Keywords:** Cardiovascular prevention, Lifestyle intervention programmes, Cardiovascular risk

## Abstract

**Background:**

Cardiovascular disease is a major cause of mortality and morbidity worldwide, and primary prevention efforts are poorly developed in people at high cardiovascular risk. On this background, we performed the Hjerteløftet Study and demonstrated that participation over 36 months in a multimodal primary prevention programme, significantly reduced validated cardiovascular risk scores. In the current substudy we aimed to further explore several elements and effects following the intervention programme.

**Methods:**

A random sample from the original Hjerteløftet Study was included for further examinations (*n* = 255, 40% women), and these patients were already randomized to an intervention group (IG) (*n* = 127), or a control group (CG) (*n* = 128). We compared changes from baseline to 36-months follow-up in physical activity, cardiorespiratory fitness, psychological well-being (WHO-5), cardiovascular medication use, smoking habits, and cardiometabolic risk factors (blood pressure, lipids, blood glucose, HbA1c, Apolipoprotein A-I, Apolipoprotein B and high-sensitive C-reactive protein).

**Results:**

Self-reported physical activity increased significantly with absolute difference in mean delta Physical Activity Index score in the IG compared to the CG: 0.90, 95% CI: 0.10 to 1.70, *p* = 0.028 (ANCOVA). There were no corresponding differences in cardiorespiratory fitness. The participation resulted in psychological well-being improvement in both groups with a larger increase in the IG compared to the CG. The mean difference in delta WHO-5 score was 5.06, 95% CI: 0.68 to 9.45, *p* = 0.024, and 3.28, 95% CI: -0.69 to 5.25, *p* = 0.104 when controlled for baseline values (ANCOVA). The use of antihypertensive medication increased significantly more in the CG (*p* = 0.044). Only minor, nonsignificant changes were observed for traditional risk factors and cardiometabolic variables.

**Conclusions:**

Participation in the Hjerteløftet Study intervention programme resulted in an improved physical activity level, but without changing cardiorespiratory fitness. Participation in the programme also tended to improve psychological well-being, possibly related to increased physical activity, less smoking and less use of cardiovascular medication. Concerning the metabolic status, no major differences were observed, but minor changes may have been concealed by a larger increase in cardiovascular medication use in the control group.

**Trial registration:**

ClinicalTrials.gov (NCT01741428), 04/12/2012.

## Introduction

Cardiovascular diseases (CVD) remain a leading cause of premature mortality and morbidity in large parts of the world and are primarily caused by lifestyle-related risk factors that may be preventable through healthy lifestyle and risk factor control [1].

On this background we conducted the Hjerteløftet Study, a randomized controlled trial to examine effects of a multimodal intervention programme including a hospital-based lifestyle course and primary care follow-up over 36 months, in individuals with elevated cardiovascular risk [2]. The intervention resulted in a significant lowering of several validated risk scores (NORRISK, FRAMINGHAM, PROCAM and NORRISK2), when compared to usual care [2]. Physical activity (PA), stress management, nutrition and smoking cessation were major elements in the programme, and thus may in variable degree all have contributed to the observed reduction in risk scores.

PA is a cornerstone in cardiovascular prevention as it is associated with reduced risk of CV morbidity and mortality, all cause mortality as well as incidence of type 2 diabetes mellitus [3]. Physical inactivity is an independent risk factor for future CVD [4]. For physically inactive adults, even light-intensity PA is likely to produce health benefits [1]. To modify and maintain lifestyle habits, including PA, is challenging, however. An important aim in the Hjerteløftet intervention programme was to expose the individuals to a wide range of different activities in order to increase the likelihood of finding activities to continue over time, as an increase in PA and improvement in cardiorespiratory fitness (CRF) both are strongly associated with reduction in future CVD [5, 6].

Associations between adverse psychological factors, such as depression and anxiety, and CVD are well-established [1]. Further, accumulating evidence suggests that psychological well-being, which includes positive thoughts and feelings such as purpose in life, optimism, and happiness, has independent associations with lower risk of CVD, and may thus promote cardiovascular health [7]. A major focus in the Hjerteløftet intervention programme was empowering the individuals in taking care of their health, through providing opportunities to develop mental and practical skills, including stress management.

Many biomarkers have been found to be linked to CVD risk and are suggested to improve risk stratification above classical risk factor evaluation. These biomarkers may reflect additional underlying mechanisms, including inflammation and subclinical disturbances in glucose metabolism [8, 9].

In this substudy of the Hjerteløftet Study we aimed to further explore several elements and effects of the intervention programme, including changes in physical activity, cardiorespiratory fitness, psychological well-being, as well as a number of metabolic parameters related to lipids, inflammation and glucose metabolism.

## Methods

### Patients and setting

**Fig. 1:**
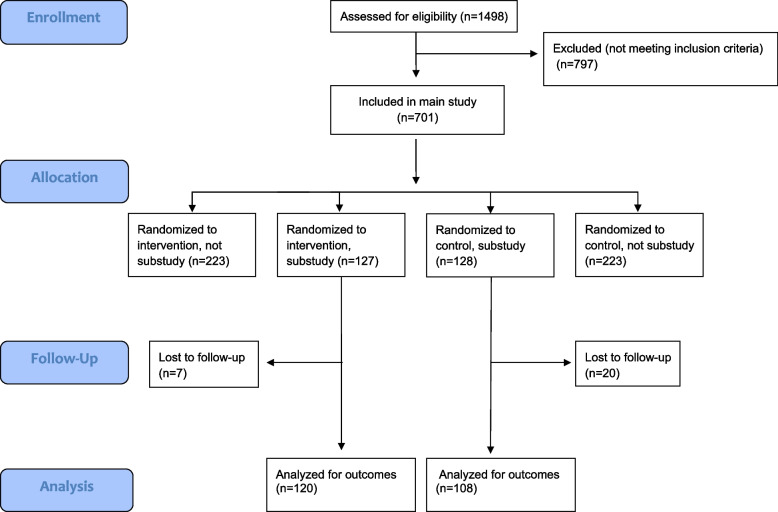
The CONSORT flow diagram

As described in detail previously [2], patients in the Hjerteløftet Study were referred for participation from general practitioners (GP) in Norway and could be included if 35—67 years of age and had elevated CVD risk. Elevated risk was defined as being at least 50% of the risk-threshold where pharmacological intervention was recommended according to national clinical guidelines [10]. In the same procedure as the patients were randomized to intervention or to control in the main study (1:1), the patients were also randomized for participation or not in the substudy (1:1). The present study population hence represents a random sample from both arms of the original total of 701 patients who participated in the Hjerteløftet Study in the period 2011—2019 (Fig. 1). A permuted block randomization was generated, and sealed opaque envelopes with consecutive inclusion numbers were made. The sequence was generated by Oslo Centre for Biostatistics and Epidemiology, Oslo University Hospital.

### Interventions

The Hjerteløftet intervention programme is previously described [2] and is only briefly summarized here. Patients randomized to the intervention group (IG) participated in a 5-day hospital-based lifestyle course in a specialized cardiac rehabilitation hospital (CR Hospital) which focused on PA, nutrition, smoking cessation, stress-management, motivation and individual goal-setting. They received information about their CVD risk and were given guideline-based recommendations for lifestyle changes and pharmacological treatment. More specific regarding the exercise intervention, the aim during the course was to educate the patients about the benefits of exercise and the safety of exertion despite the presence of cardiovascular risk factors. Based on the results of a cardiopulmonary exercise test (CPET) the patients received individual advice on recommended exercise. Exercise sessions consisted of aerobic exercises, interval training, resistance training, aqua-aerobics and attention training. The purpose of the exercise-programme was to introduce the individuals to a wide spectrum of activities and sports to make it more likely they would find activities of interest which they would be able to continue in the future. During the course the patients made a graded exercise plan for the follow-up period themselves, supported by the study staff, tailored to the patient`s lifestyle, motivation and PA level. The patients were encouraged to provide overview of local PA opportunities and make appointments before leaving the course.

### Outcomes and assessments

All outcome measurements were performed at baseline and after 3 years by the study staff at the CR Hospital. At baseline, the patients in the IG performed the measurements during the lifestyle course, while the patients in the control group (CG) attended a one-day assessment at the CR Hospital. Both groups performed the follow-up measurements during a one-day visit at the CR Hospital.

#### Cardiopulmonary exercise testing

CRF was assessed, using a Schiller Ganshorn ergo-spirometry system (Schiller AB, Baar, Switzerland) at baseline, and with a Wyntus CPX (Customed, Ottobunn, Germany) or the Schiller Ganshorn ergo-spirometry system at follow-up by direct measurement of oxygen consumption walking or running on a treadmill. Participants performed an 8-min warm up followed by a stepwise protocol chosen by experienced test personal. The patients were strongly encouraged to exercise to exhaustion. Respiratory exchange ratio ≥ 1.1, or that VO_2_ did not increase (more than 2 mL/kg/min) despite increased workload, was used to verify maximal effort. VO_2peak_ was defined as the highest oxygen uptake averaged over 30 s and are presented as absolute (L/min) and relative (mL/kg/min) values. Furthermore, the CPET was performed with continuous 12-lead electrocardiogram monitoring.

#### Self-reported physical activity

Leisure-time physical activity was assessed using a three-item questionnaire capturing frequency, intensity and duration [11]. Frequency was assessed as “How frequently do you exercise?” with the response options: “Never” [0], “Less than once a week” [0], “Once a week” [1], “2–3 times per week” [2.5] and “Almost every day” [5]. Intensity was assessed as “If you exercise as frequently as once or more times a week: How hard do you push yourself?” with the response options: “I take it easy without breaking a sweat or losing my breath” [1], “I push myself so hard that I lose my breath and break into sweat” [2] and “I push myself to near exhaustion” [3]. Duration was assessed as “How long does each session last?” with response options: “Less than 15 min” [0.1], “15–29 min” [0.38], “30 min to 1 h” [0.75] and “More than 1 h” [1.0]. A Physical Activity Index (PAI) score was calculated for each patient by multiplying each patient’s response to the above three questions, i.e. numbers in claims []. The patients were classified into three categories based on the score-values. An index score ranging 0.05 – 1.50 was considered to indicate low activity, a score ranging 1.51 – 3.75 indicated medium activity and a score ranging 3.76 – 15.00 high activity. The questionnaire has previously been validated against objective measurement methods and the International Physical Activity Questionnaire, with good internal consistency [11].

#### Accelerometry measured physical activity

PA was also measured using the ActiGraph GT3X + accelerometer (ActiGraph, LLC, Pensacola, Florida, USA). The ActiGraph instrument has been validated and will collect data on PA from sedentary to very vigorous [12]. The patients were instructed to wear the accelerometer over the hip for seven consecutive days and only to remove it at night and during water activities. Accelerometer data were accepted if the patient wore the monitor minimum 10 h a day for at least four days. The output from the accelerometer includes total PA (mean counts per minute), minutes in sedentary time and minutes in light, moderate and vigorous physical activity using standard cut-off points [13].

#### Psychological well-being

The patients` experiences related to psychological well-being were assessed using the five-item World Health Organization Well-Being Index (WHO-5), which is among the most widely used questionnaires assessing subjective psychological well-being [14]. The short questionnaire is reported to have adequate validity as an outcome measure in clinical trials. It has shown to be able to capture improvements in well-being caused by pharmacological and non-pharmacological interventions, and it has been applied successfully across a wide range of study fields. The questionnaire contains the following items (1) “I have felt cheerful and in good spirits”, (2) “I have felt calm and relaxed”, (3) “I have felt active and vigorous”, (4) “I woke up feeling fresh and rested” and (5) “My daily life has been filled with things that interest me”. The patient is asked to rate how well each of the statements applies to her or him when considering the last 14 days. Each of the 5 items is scored from 0 (none of the time) to 5 (all the time) and a raw score therefore ranges from 0 (absence of well-being) to 25 (maximal well-being). The raw score is multiplied with 4, translating the scale into a percentage scale from 0 (absent) to 100 (maximal).

#### Demographic data, smoking and cardiovascular medication

Demographic data on educational level, employment status, smoking status and cardiovascular medication were collected via questionnaire, and were checked while interviewing the patient in the hospital and then compared with information given from the patient`s GP.

#### Weight, Body Mass Index, waist circumference

Waist circumference was measured in centimetres (cm) at the umbilical level with a measuring tape. Body weight was measured without shoes, wearing exercise clothes prior to CPET. Body Mass Index (BMI) was calculated by dividing body weight by body height squared (m^2^).

#### Cardiometabolic risk factors

Fasting venous blood samples were analysed for triglycerides (Tg), low-density lipoprotein (LDL), high-density lipoprotein (HDL), total cholesterol (TC), Apolipoprotein A-I (ApoA1), Apolipoprotein B (ApoB), high-sensitive C-reactive protein (hs-CRP), glycosylated haemoglobin (HbA1c) and insulin at Department of Laboratory Medicine at the Oslo University Hospital, Rikshospitalet. Blood pressure (BP) was measured manually, preferably on the left upper arm with a proper sized cuff and repeated three times with one-minute intervals after five minutes rest, of which the mean of the two last readings was used in the analysis.

### Follow-up

The 3-year follow-up period was allocated to primary care and included a periodic digital follow-up option from the hospital. Follow-up was based on each patient`s individual plan for achieving and maintaining risk factor control decided at the lifestyle course. Relevant follow-up contacts, in addition to the GPs, were Healthy Life Centers, a personal mentor, fitness centres, voluntary services etc. Regarding exercise, both the type of activity, the frequency, intensity and the duration of the activity sessions during the follow-up period were decisions made by the patient. Recommendations for follow-up intervals by the GPs were given, but the actual follow-up routine, including any need for adjustments in cardiovascular medication, was left to the GP. The CG received care as usual in the community health care by the GP.

### Statistical methods

IBM SPSS Statistics (version 29) was used for statistical analysis. Categorical variables were summarized as frequencies and continuous variables by mean with its standard deviation. The paired samples t-test was used to analyse within-group differences from baseline to follow-up for continuous variables as was the McNemars`s test for categorical variables. Outcome measures consisting of continuous variables were analysed for differences between groups using Student t-test and to control for baseline imbalance we also used analysis of covariance (ANCOVA). Outcome measures consisting of categorical variables were analysed for differences between groups using Mann Whitney test. A *p*-value < 0.05 was considered significant. All analyses were perfomed on patients with complete follow-up data, considering the explorative nature of the study. No specific power calculation was performed ahead of study for the substudy variables, as the primary outcome in the main study was change in total cardiovascular risk, on which power calculations were based.

### Ethics

This study was approved by the Regional Committee for Medical Health Research Ethics (ID: 2011/561a) as a substudy of the previously described Hjerteløftet Study [2]. All included patients provided written informed consent. The study protocol was registered in ClinicalTrials.gov (NCT0174128).

## Results

A total of 255 individuals (40% women) were included in this substudy, out of the main study population of 701, whereof 127 patients from the IG and 128 from the CG. After randomization, 7 patients in the IG and 20 patients in the CG were lost to follow up. Finally, 228 (89.5%) patients remained in the follow-up study population, with 120 (95%) patients in the IG and 108 (84%) in the CG (Fig. 1). Baseline characteristics are presented in Table 1 and 2.

**Table 1:** Baseline demographic and clinical characteristics

Baseline variables	Total sample (*n* = 255)	Intervention (*n* = 127)	Control (*n* = 128)
Mean age (SD), years	54.6 (8.1)	54.6 (8.7)	54.5 (7.6)
Sex			
Female	103 (40%)	47 (37%)	56 (44%)
Current employed	169 (67%)	84 (67%)	85 (66%)
Education level			
Primary school	37 (15%)	18 (15%)	19 (15%)
High school	110 (45%)	62 (50%)	48 (39%)
College	99 (40%)	43 (35%)	56 (46%)
Current smokers	78 (31%)	39 (31%)	39 (31%)
Metabolic syndrome	155 (67%)	80 (67%)	75 (67%)
Diabetes mellitus	42 (17%)	21 (16%)	21 (17%)
Mean blood pressure (SD)			
Systolic (mmHg)	133 (14)	132 (14)	133 (15)
Diastolic (mmHg)	84 (10)	83 (10)	85 (10)
Mean blood values (SD)			
Total cholesterol (mmol/l)	5.16 (1.11)	5.10 (1.08)	5.23 (1.14)
LDL-cholesterol (mmol/l)	3.23 (0.99)	3.12 (1.00)	3.35 (0.96)
HDL-cholesterol (mmol/l)	1.21 (0.39)	1.19 (0.42)	1.24 (0.36)
Triglycerides (mmol/l)	1.72 (1.32)	1.90 (1.70)	1.53 (0.68)
Fasting blood glucose (mmol/l)	6.49 (1.62)	6.52 (1.64)	6.46 (1.60)
HbA1c (%)	5.99 (0.81)	6.03 (0.85)	5.95 (0.78)
Insulin (pmol/l)	100.42 (102.16)	97.91 (70.21)	103.12 (128.14)
ApoA1 (g/l)	1.48 (0.29)	1.46 (0.29)	1.49 (0.29)
ApoB (g/l)	1.01 (0.27)	1.00 (0.29)	1.01 (0.24)
hsCRP (mg/l)	3.05 (3.30)	2.99 (3.21)	3.12 (3.39)
Waist circumference (SD), cm	106.7 (14.1)	106.8 (13.6)	106.7 (14.8)
Men	110.4 (12.5)	109.4 (12.2)	111.6 (12.9)
Women	101.3 (14.7)	102.4 (14.7)	100.2 (14.8)
BMI (SD),kg/m^2^	30.9 (5.4)	30.9 (5.2)	30.9 (5.6)
Men	31.4 (5.0)	31.0 (4.8)	31.8 (5.1)
Women	30.1 (5.9)	30.7 (5.7)	29.6 (6.0)
Weight (SD), kg	93.8 (20.7)	93.4 (19.4)	94.3 (22.2)
Men	101.7 (18.9)	99.9 (18.4)	103.8 (19.4)
Women	82.1 (17.7)	82.6 (16.0)	81.6 (19.3)
Cardiovascular medication			
Antihypertensive drugs	134 (52.5%)	67 (52.8%)	67 (52.3%)
Lipid-lowering drugs	94 (36.9%)	53 (41.7%)	41 (32.0%)
Antidiabetic medication	29 (11.4%)	16 (12.6%)	13 (10.2%)

*SD* standard deviation, *LDL-cholesterol* low-density lipoprotein cholesterol, *HDL-cholesterol* high-density lipoprotein cholesterol, *HbA1c* glycosylated hemoblobin, *ApoA1* apolipoprotein A1, *ApoB* apolipoprotein B, *hsCRP* high sensitivity C-reactive protein, *BMI* body mass index

Data are n (%) or mean (SD) unless stated otherwise

**Table 2:** Self-reported physical activity, accelerometry measured physical activity, physical fitness and psychological well-being at baseline

**Baseline variables**	Total (*n* = 255)	Intervention (*n* = 127)	Control (*n* = 128)
**Self-reported physical activity**			
Physical Activity Index (PAI)	2.90 (2.64)	2.56 (2.40)	3.27 (2.85)
Inactive (PAI 0–0.04)	44 (19.3%)	24 (20.3%)	20 (18.2%)
Low physical activity (PAI 0.05–1.5)	49 (21.5%)	30 (25.4%)	19 (17.3%)
Medium physical Activity (PAI 1.51–3.75)	81 (35.5%)	43 (36.4%)	38 (34.5%)
High physical activity (PAI 3.76–15)	54 (23.7%)	21 (17.8%)	33 (30%)
**Accelerometry measured physical activity**			
Cpm (counts per minute)	286.9 (105.2)	274.8 (106.6)	299.9 (102.9)
Sedentary time (minutes/day)	655.3 (86.3)	663.2 (87.9)	646.9 (84.3)
Light physical activity (minutes/day) |	185.6 (53.6)	188,1 (53.4)	183.0 (54.0)
Moderate physical activity (minutes/day)	37.6 (19.6)	36.9 (20.6)	38.4 (18.6)
Vigorous physical activity (minutes/day)	1.2 (3.3)	0.9 (1.8)	1.6 (4.3)
**Cardiorespiratory exercise testing**			
VO_2peak_ (ml/kg/min)	29.1 (6.4)	29.0 (6.7)	29.2 (6.1)
VO_2peak_ (l/min)	2.66 (0.8)	2.66 (0.7)	2.67 (0.8)
RER_peak_	1.19 (0.10)	1.17 (0.09)	1.21 (0.11)
VO_2peak_ (ml/kg/min), men	31.7 (5.6)	32.0 (5.7)	31.4 (5.4)
VO_2peak_ (l/min), men	3.15 (0.6)	3.12 (0.5)	3.19 (0.7)
VO_2peak_ (ml/kg/min), women	25.2 (5.5)	24.0 (4.8)	26.3 (5.8)
VO_2peak_ (l/min), women	1.95 (0.3)	1.91 (0.3)	1.98 (0.3)
**Psychological well-being**			
WHO-5 standardized %- score	61.8 (17.9)	58.8 (18.1)	65.0 (17.2)

*SD* standard deviation, *VO*_*2peak*_ peak oxygen consumption, *RER* respiratory exchange ratio

Data are mean (SD) or n (%) unless stated otherwise

### Physical activity

**Fig. 2:**
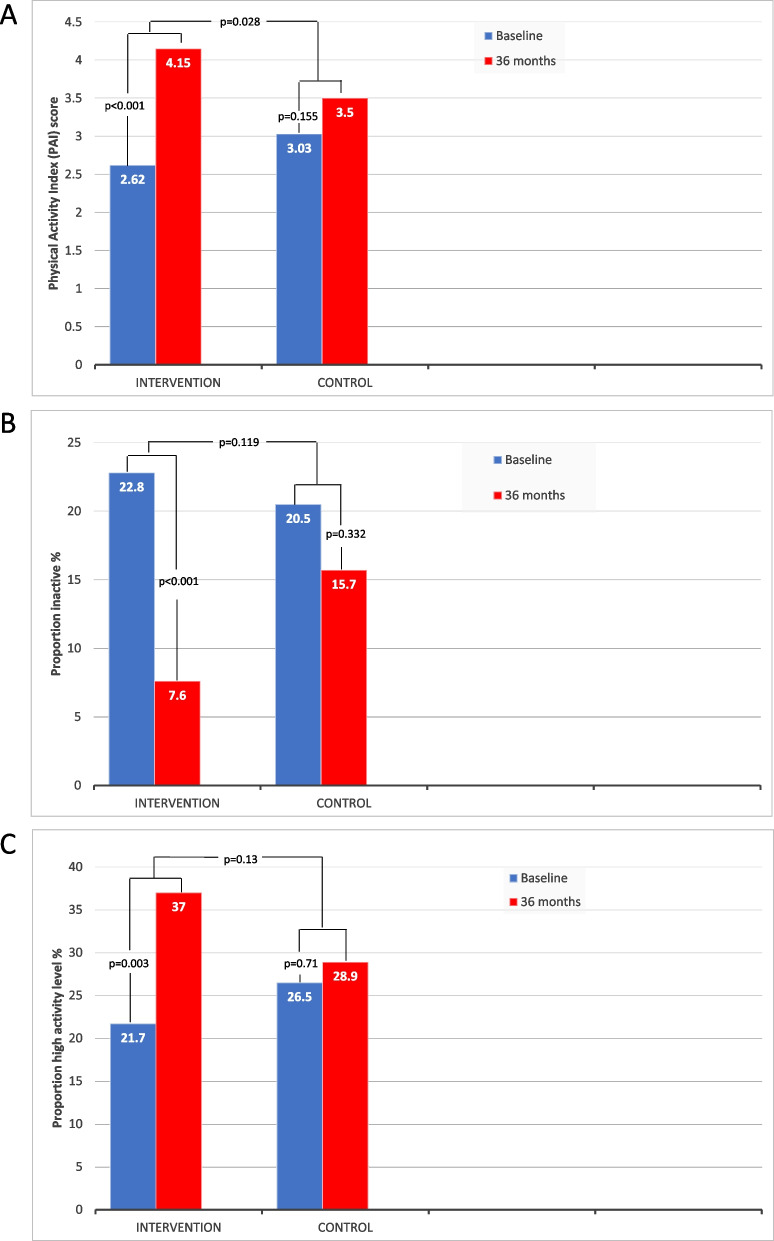
Changes in physical activity level from baseline to 36 months follow-up**. a**) Physical Activity Index (PAI) score **b**) Proportion inactive patients **c**) Proportion patients performing high activity

PA levels increased significantly more in the IG compared to the CG during follow-up. The difference between the mean change PAI scores was 1.07, 95% CI: 0.21 to 1.93, *p* = 0.015 on intervention vs usual care. The difference remained significant when controlling for baseline imbalance using ANCOVA; 0.90, 95% CI: 0.10 to 1.70, *p* = 0.028, (Table 3, Fig. 2a). There also was a within group significant decrease in the proportion being inactive in the IG from baseline to follow-up (Fig. 2b), and the proportion performing high activity was significantly increased (Fig. 2c). Accelerometer registrations showed a total physical activity of 287 counts per minute (cpm) at baseline and that the population spent 655 min sedentary a day, where men being more sedentary (i.e. 60 min a day) than women. Statistical analyses to assess changes between baseline and follow-up were not considered appropriate because a large proportion of non-valid registrations at follow-up.

Concerning CRF, no differences between groups were observed during intervention (Table 3).

**Table 3:** Changes in physical activity behaviour, physical fitness and psychological well-being in patients receiving intervention vs usual care from baseline to 36 months follow-up

	**Mean (SD)/Proportion**			
	**Intervention** (*n* = 120)	**Control** (*n* = 108)	Difference between means (95% CI)/proportions	*p*-value
**PAI score**				
Baseline	2.62 (2.56)	3.03 (2.61)		
Follow-up	4.15 (3.03)	3.50 (3.08)		
Change, *p*-value	1.53 (2.82), < 0.001^c^	0.46 (2.93), 0.155^c^	1.07 (0.21 to 1.93)	0.015^a^
ANCOVA			0.90 (0.10 to 1.70)	0.028
**Activity level**				
** Inactive** (PAI 0–0.04)				
Baseline	21 (22.8%)	17 (20.5%)		
Follow-up	7 (7.6%)	13 (15.7%)		
Change, *p*-value	-14 (15.2%), < 0.001^d^	-4 (4.8%), 0.332^d^	10.4%	0.119^b^
** Low activity** (PAI 0.05–1.5)				
Baseline	23 (25%)	13 (15.7%)		
Follow-up	15 (16.3%)	15 (18.1%)		
Change, *p*-value	-8 (8.7%), *p* = 0.144^d^	2 (2.4%), 0.617^d^	11.1%	0.144^b^
** Medium activity** (PAI 1.51–3.75)				
Baseline	28 (30.4%)	31 (37.3%)		
Follow-up	36 (39.1%)	31 (37.3%)		
Change, *p*-value	8 (8.7%), 0.157^d^	0	8.7%	0.414^b^
** High activity** (PAI 3.76–15)				
Baseline	20 (21.7%)	22 (26.5%)		
Follow-up	34 (37%)	24 (28.9%)		
Change, *p*-value	14 (15.3%), 0.003^d^	2 (2.4), 0.71^d^	12.9%	0.130^b^
** VO**_**2peak**_ ml/kg/min				
Baseline	29.0 (6.7)	29.2 (6.1)		
Follow-up	28.4 (6.6)	28.7 (7.2)		
Change, *p*-value	-0.7 (4.2), 0.117^c^	-0.5 (6.4), 0.443^c^	-0.1 (-1.67 to 1.39)	0.854^a^
ANCOVA			-0.2 (-1.63 to 1.26)	0.803
** VO**_**2peak**_ l/min				
Baseline	2.67 (0.7)	2.67 (0.8)		
Follow-up	2.64 (0.7)	2.63 (0.9)		
Change, *p*-value	-0.04 (0.4), 0.344^c^	-0.04 (0.5), 0.397^c^	-0.01 (-0.11 to 0.13)	0.927^a^
ANCOVA			-0.01 (-0.11 to 0.12)	0.915
** WHO5 standardized %score**				
Baseline	59.78 (17.96)	64.65 (17.22)		
Follow-up	69.14 (17.62)	68.95 (16.34)		
Change, *p*-value	9.36 (15.33), < 0.001^c^	4.30 (13.22), 0.005^c^	5.06 (0.68 to 9.45)	0.024^a^
ANCOVA			3.28 (-0.69 to 5.25)	0.104

*ANCOVA* analysis of covariance, *CI* confidence interval, *SD* standard deviation, *PAI score* Physical Activity Index Score

^a^Comparison between the two groups by Students t-test

^b^Comparison between groups by Mann–Whitney test

^c^Change within group by paired t-test

^d^Change within group by McNemars test

### Psychological well-being

**Fig. 3:**
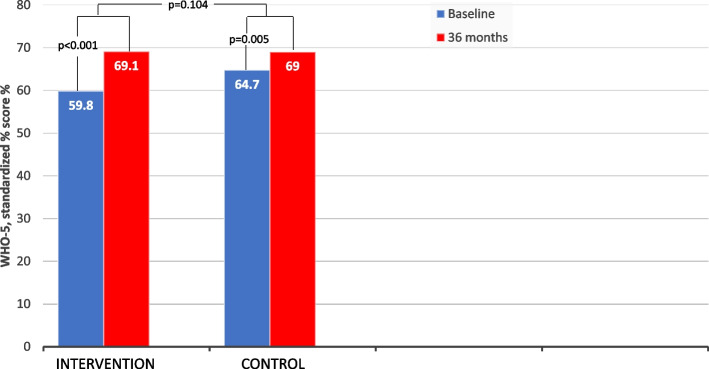
Changes in WHO-5 score from baseline to 36 months follow-up

The WHO-5 score increased in both groups during follow-up (Table 3, Fig. 3). The difference between mean change scores indicated a significant increase of 5.06, 95% CI: 0.68 to 9.45, *p* = 0.024, on intervention vs. control. When controlling for baseline imbalance using ANCOVA, the difference between the mean change scores of each treatment group was 3.28 and no longer significant (95%CI: 0.68 to 9.45, *p* = 0.104).

### Cardiovascular risk factors and cardiometabolic variables

Data on cardiovascular risk factors are shown in Table 4. Regarding cardiometabolic variables the records showed a within group significant reduction in HbA1c in the IG from baseline to follow-up. Otherwise only minor changes were observed for traditional CV risk factors and metabolic variables, and no significant differences between groups. The proportion of individuals who quit smoking in the IG was numerically higher than in the CG (*n* = 17 vs *n* = 9).

**Table 4:** Changes in cardiometabolic risk factors and cardiovascular medication in patients receiving intervention vs usual care from baseline to 36 months follow-up

	Intervention (*n* = 120)	Control (*n* = 108)	Difference between means (95% CI)/proportions	*p*-value (between groups)
Antihypertensive medication				
Baseline	64 (53.3%)	57 (52.8%)		
Follow-up	67 (55.8%)	70 (64.8%		
Change, *p*-value	3 (2.5%), 0.405^c^	13 (12%), 0.002^c^	10 (9.5%)	0.044^b^
Lipid-lowering medication				
Baseline	51 (42.5%)	34 (31.5%)		
Follow-up	57 (47.5%)	48 (44.4%)		
Change, *p*-value	6 (5%), 0.109^c^	14 (12.9%), 0.002^c^	8 (7.9%)	0.106^b^
Antidiabetic medication				
Baseline	14 (11.7%)	11 (10.2%)		
Follow-up	19 (15.8%)	18 (16.7%)		
Change, *p*-value	5 (4.1%), 0.059^c^	7 (6.5%), 0.008^c^	2 (2.4%)	0.479^b^
Daily smokers				
Baseline	36 (30%)	31 (28.7%)		
Follow-up	19 (15.8%)	22 (20.4%)		
Change, *p*-value	17 (14.2%), < 0.001^c^	9 (8.3%), < 0.003^c^	8 (5.9%)	0.176^b^
SBP (mmHg)				
Baseline	131.95 (13.58)	132.82 (16.07)		
Follow-up	133.66 (15.83)	133.08 (14.0)		
Change, *p*-value	1.71 (15.39), 0.286^d^	0.26 (15.46), 0.869^d^	1.39 (-2.97 to 5.74)	0.531^a^
ANCOVA			-0.92 (-2.83 to 4.68)	0.628
DBP (mmHg)				
Baseline	81.88 (8.80)	84.79 (10.0)		
Follow-up	83.61 (8.88)	83.07 (8.68)		
Change, *p*-value	1.73 (9.60), 0.075^d^	-1.72 (10.33), 0.109^d^	3.45 (0.63 to 6.26)	0.017^a^
ANCOVA			1.63 (-0.68 to 3.93)	0.166
Total cholesterol (mmol/l)				
Baseline	5.06 (1.06)	5.28 (1.18)		
Follow-up	5.07 (1.10)	5.05 (1.26)		
Change, *p*-value	0.01 (1.19), 0.933^d^	-0.23 (1.33), 0.101^d^	0.24 (-0.12 to 0.59)	0.194^a^
ANCOVA			0.11 (-0.20 to 0.41)	0.492
Weight (kg)				
Baseline	92.86 (19.28)	92.94 (21.73)		
Follow up	93.14 (19.80)	92.01(20.48)		
Change, *p*-value	0.28 (5.60), 0.613^d^	-0.93 (8.14), 0.269^d^	1.21 (-0.75 to 3.18)	0.225^a^
ANCOVA			1.21 (-0.72 to 3.13)	0.217
Waist circumference (cm)				
Baseline	106.4 (13.67)	105.9 (14.58)		
Follow-up	105.5 (13.77)	105.5 (14.49)		
Change, *p*-value	-0.8 (6.24), 0.184^d^	-0.4 (8.41), 0.648^d^	-0.44 (-2.52 to 1.64)	0.677^a^
ANCOVA			-0.38 (-2.40 to 1.64)	0.711
BMI (kg/m^2^)				
Baseline	30.80 (5.25)	30.58 (5.41)		
Follow-up	30.77 (5.48)	30.27 (5.37)		
Change, *p*-value	-0.03 (1.91), 0.880^d^	-0.31 (2.57), 0.248^d^	0.28 (-0.36 to 0.91)	0.392^a^
ANCOVA			0.29 (-0.34 to 0.92)	0.361
Fasting blood glucose (mmol/l)				
Baseline	6.44 (1.59)	6.33 (1.45)		
Follow-up	6.42 (1.45)	6.29 (1.60)		
Change, *p*-value	-0.02 (1.12), 0.871^d^	-0.04 (1.11), 0.762^d^	0.02 (-0.30 to 0.34)	0.915^a^
ANCOVA			0.05 (-0.25 to 0.35)	0.753
HbA1c (%)				
Baseline	6.01 (0.84)	5.91 (0.71)		
Follow up	5.87 (0.78)	5.83 (0.82)		
Change, *p*-value	-0.14 (0.42), 0.001^d^	-0.08 (0.42), 0.055^d^	-0.06 (-0.18 to 0.06)	0.342^a^
ANCOVA			-0.05 (-0.16 to 0.07)	0.439
Insulin (pmol/l)				
Baseline	89.97 (60.32)	103.28 (137.58)		
Follow-up	97.65 (64.24)	84.31 (50.27)		
Change, *p*-value	7.68 (67.13), 0.255^d^	-18.98 (136.06), 0.177^d^	26.66 (-3.42 to 56.73)	0.082^a^
ANCOVA			15.21 (-0.67 to 31.09)	0.060
hsCRP (mg/l)				
Baseline	2.84 (3.04)	2.90 (2.95)		
Follow-up	2.72 (3.12)	2.90 (5.21)		
Change	-0.13 (3.43), 0.714^d^	0.00 (4.54)	-0.13 (-1.26 to 1,00)	0.824^a^
ANCOVA			-0.15 (-1.24 to 0.94)	0.784

^a^Comparison between groups by Students t-test

^b^Comparison between groups by Mann-Whitney test

^c^Change within group by McNemars test

^d^Change within group by paired t-test

### Cardiovascular medication

**Fig. 4:**
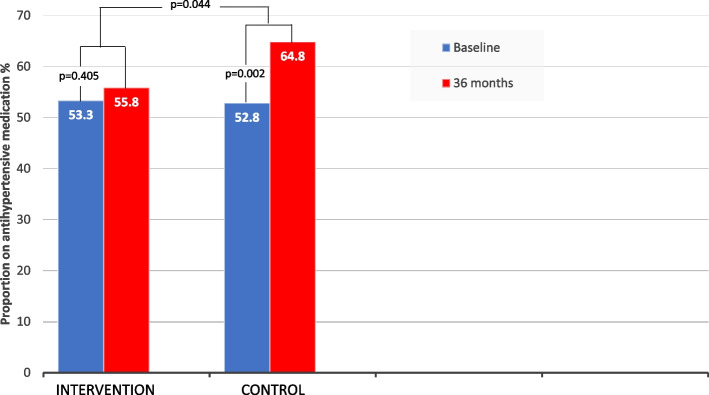
Changes in the proportion of patients on antihypertensive medication from baseline to 36 months follow-up

53% of the patients in both groups used antihypertensives when included in the study, and the proportion of patients using lipid-lowering and glucose-lowering medication was also high (Table 1). Cardiovascular medication use increased in both groups during follow-up and there was a within group significant increase in both lipid-lowering drugs, antihypertensives and glucose-lowering drugs in the CG at 3 years, which was less prominent in the IG (Table 4). As regards the use of antihypertensives during follow-up, we observed a significant larger increase in the CG vs the IG (*p* = 0.044), (Fig. 4).

## Discussion

### Physical activity and cardiorespiratory fitness

A major finding in the present substudy of a primary prevention trial [2], was a statistically significant increased PA level in favour of the IG. The program also resulted in a within group significant decrease in the proportion of patients reporting being inactive in the IG, while the decrease was less prominent in the CG. Objective measurements of PA showed that baseline sedentary time was 10.9 h/day (Table 2), indicating substantial need for improvement. Unfortunately, limited valid registrations prohibited definitive confirmation of the self-reported findings. In contrast, the records showed that there were no changes in CRF in either group during 3 years of follow-up, and no differences between groups.

The questionnaire used to assess PA, was the same as the one used in HUNT 1 and HUNT 3 [11], making it possible to compare PA levels to the general Norwegian population. Hence, it seems fair to conclude that the intervention was able to promote positive changes as regards PA levels, but without improving CRF.

Multiple lifestyle trials aiming sustainable lifestyle changes have shown a varying degree of improvements in PA and CRF. Few trials have follow-up time as long as 3 years. However, in such trials there seem to be a pattern of transient favourable changes that dilute over time, and that sustainability may depend on the maintenance of the coaching support and the intensity and the duration of the follow-up intervention [15–17].

Even if a significant increased PA level was observed in the IG, the improvements were not sufficient to increase CRF. Regular PA is an effective means of increasing CRF and CRF improvement is related to both intensity, duration and frequency of the activity [18]. The optimal exercise prescription for most efficient CRF improvements remain to be clearly defined. There is evidence that high-intensity exercise training performed as aerobic interval training is more effective in improving CRF than moderate- and low-intensity training in both healthy individuals [18], in patients with metabolic syndrome [19], coronary heart disease [20] and overweight and obese individuals [21]. Less sedentary behaviour has also in some studies been shown to have the potential to improve CRF, even if the results in different studies are mixed [22]. In the Hjerteløftet Study no specific training protocol was given concerning intensity or amount of exercise as the patients made their own plan based on their preferences and opportunities in their home-situation. Thus, we have limited knowledge concerning the exact mode of increase in activity in our study, other than that the PAI score, which includes both intensity, frequency and duration, was significantly increased, reflecting an overall improvement in PA level. Even if CRF was not increased in the IG in our study, the increased PA level may have contributed to the maintenance of CRF during 3 years of aging, as CRF otherwise would be expected to decline progressively with age [23]. Maintenance of CRF in the CG during follow-up, where only a marginal increase in PA was observed, may be related to that also CG patients could have been motivated to exercise more in advance of the 3-year assessment which they knew included a CPET.

The effect profile of our intervention programme should be seen in the light of the intensity and duration of the intervention given. The Hjerteløftet intervention programme was designed as part of a low-cost, pragmatic trial, enabling it to be carried through in a large scale. Nevertheless, the patients reported that they were significantly more physically active 3 years after the lifestyle course in the beginning of the study, compared to controls.

Improvement of CRF may appear as an obvious target in cardiovascular prevention as higher levels of CRF is associated with favourable levels of CVD risk factors. On the other hand, a large and growing number of studies on health benefits of PA show that a change from physical inactivity to lower or moderate activity is the most important relative step for disease prevention [24–26]. A recent meta-analysis investigating associations between non-occupational PA and risk for CVD, cancer and mortality outcomes found substantial protection against a range of chronic disease outcomes, including CVD, from small increases in non-occupational PA in inactive adults [27].

Emerging evidence indicates that a daily sedentary time exceeding 9.5 h/day is associated with increased risk for CVD morbidity and mortality, even after accounting for traditional risk factors [28, 29]. The replacement of sedentary time with even light and/or moderate-to-vigorous PA has large potential to improve CVD risk [30, 31]. A recent meta-analysis by Eklund et al., which examined the association between PA, sedentary time and mortality, showed that higher levels of PA, at any intensity, and less time spent sedentary, were associated with substantially reduced risk for premature mortality [32]. In our study, accelerometer-measured sedentary time of 10.9 h/day at baseline exceeded the average Norwegian population level of 9.2 h/day [33]. This may have contributed to the patients` baseline elevated risk, in a similar manner as their reduced CRF level compared to the general healthy Norwegian population [34]. The oxygen uptake was 79% and 92% of the general population level in ml/kg/min and l/min, respectively.

Our findings of less inactivity and corresponding increased proportion of patients performing medium and high activity, may support that important CVD risk reduction may be widely achievable through reducing sedentary behaviour [35]. The mechanisms by which reduced sedentary time improve cardiovascular risk are, however, incompletely understood and may only in part be apparent in the risk factors included in most risk score algorithms. From our data, a reduction in metabolic syndrome and need for antihypertensive medication may link the observed reduction in sedentary time to our findings of reduced cardiovascular risk scores.

### Psychological well-being

An interesting finding in our substudy was that WHO-5 score improved to above general population level in both groups during follow-up. This increase was larger in the IG, but the difference between groups did not reach significance (*p* = 0.10) when controlling for baseline imbalance. Nevertheless, our data seem to support that participation in the programme tended to improve psychological well-being. Our findings are in line with a recent systematic review reporting that healthy lifestyle interventions, targeting physical outcomes, are associated with psychological well-being improvements [36]. This is of relevance, as psychological well-being is an important health outcome by itself, but also as psychological well-being may influence CVD risk favourably, likely through both biological, behavioural as well as psychological pathways [7]. The interaction between exercise and well-being is likely bidirectional, as evidence indicates that high levels of well-being are associated with greater likelihood of regular exercise [37], which is one example of how well-being may influence CV health through supporting healthy behaviour [38]. Further, evidence shows that regular exercise can improve psychological predictors of well-being such as social relationships, identity and sense of belonging [39]. Our findings of positive effects on psychological well-being may be important when cost-effectiveness of such lifestyle interventions is considered, because of the potential effects of psychological well-being on health across a wide range of mental and physical health outcomes [40–42].

### Cardiovascular risk factors, medication and cardiometabolic variables

In the present study, only minor changes were observed regarding major cardiovascular risk factors and cardiometabolic variables, and without significant differences between groups (Table 4). Neither lipids nor their corresponding apolipoproteins did show any significant changes in any of the groups. Also, regarding BP, no significant differences were observed within or between groups. The reasons for lack of efficacy are unclear, but may be related to the intensity of the intervention, the effectiveness of the given lifestyle advice and to the well-known challenges in maintaining lifestyle changes over time. Regarding systolic blood pressure (SBP) and TC, the findings are in line with what is found in other major lifestyle studies, summarized in our recent meta-analysis assessing long-term effects of multiple lifestyle intervention in subjects with elevated CV risk [43]. In this meta-analysis we found that lifestyle intervention results in small, but significant changes in SBP and TC after 6–12 months. The benefits gradually attenuate over time, especially regarding TC, and demonstrates further reductions from 12 to 24 months. Assessments in the present study were only performed after 3-year follow-up, with no interim analyses. Possible positive intervention effects on lipids and BP may further have been camouflaged by differences in antihypertensive and lipid-lowering medication between groups, as the increase in the use of medication was higher in the CG during follow-up.

No significant differences were seen between groups regarding diabetes-related variables. We still think it is worthy pointing out a small, but significant reduction in Hba1c observed from baseline to follow-up in the IG, as Hba1c in general is expected to remain stable or increase after 3 years increase in age. This decrease cannot be ascribed to increased glucose-lowering medication. In the IG only 5 of 120 participants were started on antidiabetic drugs during the study, and the reduction in HbA1c was still significant when patients who started medication were excluded from the analysis. As body weight remained unchanged, a possible explanation for the reduction in HbA1c is the increase in PA. Dietary changes, not investigated in this study, may of course also have contributed.

The study population had a higher body weight and BMI than the general Norwegian population [23], which may have contributed to their CV risk. No significant changes were observed regarding weight, waist-circumference or BMI during follow-up. As the patients defined their own goals for behavioural change, these findings are not quite unexpected, since weight loss was a goal for only some of the patients. A further observation, as found in the main study, was that a larger proportion of patients quitted smoking in the IG compared to the CG. This may have been of relevance when patients reported improved psychological well-being [44].

Finally, we noted an interesting, reduced need for starting cardiovascular medication in the IG during the study. A high proportion of patients was already on cardiovascular medication, especially antihypertensives, and during the 3 years of follow-up, we observed a numerically larger increase in medication in the CG for both antihypertensive, glucose-lowering and lipid-lowering medication compared to the IG. Concerning antihypertensive treatment, the increased prescription trend was significantly higher in the CG. It seems possible that the higher need for starting new cardiovascular medication may have contributed to a less beneficial change in psychological well-being in the CG compared to the IG.

## Strengths and limitations

The true potential of lifestyle intervention regarding cardiovascular risk reducing effects cannot be expected to be realized in a study like the current one, as the intervention was not tightly controlled, the inclusion did not require any high commitment of the patients, and follow-up was to a large degree left to the patient and the primary care health system, already being short on resources. The general idea of the main study was to see if a low-cost intervention programme, based on the existing Norwegian health care system, could still improve cardiovascular risk scores, which was demonstrated [2]. A more intense and shorter intervention period would most likely result in larger effects of the parameters explored in the present substudy, but the generalization of the findings to public health efforts would then be more questionable.

Strengths of our study are the long follow-up time, a high patient number and a large proportion (47%) of screened individuals being included in the main study [2]. The female participation rate in our study cohort was good (40%), which is of importance for the generalizability of the findings. Another strength of the study is the careful objective measurements of CRF, that could be performed with high precision and low variability, however, only to demonstrate that CRF was not influenced by the intervention.

Our study also has some limitations. Due to the nature of the intervention, neither the study staff nor the patients were blinded for the treatment. This may have contributed to both performance and detection bias as knowledge about treatment allocation might have influenced both health care providers and the patients` behaviour. The CG also performed a one-day assessment at baseline and at follow-up in the CR Hospital, including the CPET. The follow-up and the study focus on PA, fitness and lifestyle may have motivated patients in the CG and thereby reduced the between-group difference in exercise and other favourable lifestyle changes. A limitation of the study is also the risk of attrition bias. The overall loss to follow-up was 10.6%, which may be expected in a lifestyle study with a follow-up time as long as 3 years. The difference in drop-out rates between groups, however, with more dropouts in the CG (20/128, 15.6%) than in the IG (7/127, 5.5%), may relate to that the patients wanted to participate in the study because of motivation for lifestyle change and consequently may have been disappointed when allocated to usual care. The effect of such an attrition bias is difficult to estimate, but dropouts in general have poorer outcomes than other patients, which suggests that the results in the CG could have been poorer with a more complete follow-up in this group.

Another limitation of the study was the large number of invalid accelerometer-registrations. The findings regarding PA must be considered with some caution, as analyses on changes between baseline and follow-up are based on self-reports only, and confirmation through objective measurements were hampered. Self-reports of PA are prone to recall and social desirability biases, which may result in over-estimation of PA [45, 46] and underestimation of sedentary time [47]. It should also be noted that we have limited knowledge of the adherence and the time course of the intervention effects in this study, as no interim evaluations were carried out between baseline and 3 years of follow-up. Although in-between measurements could have added interesting knowledge regarding patients` behaviour, this was not prioritized. As the main aim of the study was to assess the effects of the intervention programme on total cardiovascular risk after 3 years, interim assessments were not considered pivotal, and omitted because of resource considerations.

Finally, as any needs for adjustments in cardiovascular drug treatment was left to each patient`s GP, such adjustments may not have been implemented irrespective of the treatment allocation. It is possible that a similar study, with for example fixed lipid-lowering or antihypertensive treatment in both groups, could have detected significant group differences in these classical risk factors. In a pragmatic study over 3 years, however, it was not considered possible to avoid drug adjustments considered necessary by the patients` GP.

## Conclusions

The present substudy shows that the Hjerteløftet Study intervention programme resulted in an improved physical activity level without changing cardiorespiratory fitness. This finding may indicate that the goal of lifestyle interventions should not limit focus to promoting high-intensity training, but that important reduction in sedentary behaviour may be easier to achieve in a broad population with elevated cardiovascular risk. Our data further seem to support that participation in the programme resulted in psychological well-being improvement, with less smoking and less use of cardiovascular medication. Concerning the metabolic status, no major differences were observed, but minor changes may have been concealed by a larger increase in cardiovascular medication in the control group.

## Data Availability

The data that support the findings of this study are available from The Department of cardiac and pulmonary rehabilitation, Cathinka Guldbergs Hospital, but restrictions apply to the availability of these data, which were used under license for the current study, and so are not publicly available. Data are however available from the authors upon reasonable request and with permission of The Department of cardiac and pulmonary rehabilitation, Cathinka Guldbergs Hospital.

## Abbreviations

CVD: Cardiovascular disease
PA: Physical activity
CRF: Cardiorespiratory fitness
GP: General practitioner
IG: Intervention group
CR Hospital: Cardiac Rehabilitation Hospital
CPET: Cardiopulmonary exercise test
CG: Control group
BMI: Body mass index
Tg: Triglycerides
LDL: LDL-cholesterol
HDL: HDL-cholesterol
TC: Total cholesterol
ApoA1: Apolipoprotein A1
ApoB: Apolipoprotein B
hs-CRP: High sensitivity C-reactive protein
HbA1c: Glycosylated hemoglobin
BP: Blood pressure
ANCOVA: Analysis of covariance
Cpm: Counts per minute
SBP: Systolic blood pressure
